# Porcine lie detectors: Automatic quantification of posture state and transitions in sows using inertial sensors

**DOI:** 10.1016/j.compag.2016.07.017

**Published:** 2016-09

**Authors:** Robin Thompson, Stephanie M. Matheson, Thomas Plötz, Sandra A. Edwards, Ilias Kyriazakis

**Affiliations:** a School of Agriculture, Food and Rural Development, Newcastle University, Newcastle upon Tyne, UK; b Open Lab, Newcastle University, Newcastle upon Tyne, UK

**Keywords:** Accelerometer, Farrowing, Activity recognition, Support Vector Machine, Computational behaviour assessment

## Abstract

This paper presents a novel approach to automated classification and quantification of sow postures and posture transitions that may enable large scale and accurate continuous behaviour assessment on farm. Automatic classification and quantification of postures and posture transitions in domestic animals has substantial potential to enhance their welfare and productivity. Analysis of such behaviours in farrowing sows can highlight the need for human intervention or lead to the prediction of movement patterns that are potentially dangerous for their piglets, such as crushing when the sow lies down. Data were recorded by a tri-axial accelerometer secured to the hind-end of each sow, in a deployment that involved six sows over the period around parturition. The posture state (standing, sitting, lateral and sternal lying) was automatically classified for the full dataset with a mean *F*_1_ score (a measure of predictive performance between 0 and 1) of 0.78. Sitting was shown to present a greater challenge to classification with a *F*_1_ score of 0.54, compared to the lateral lying postures, which were classified with an average *F*_1_ score of 0.91. Posture transitions were detected with a *F*_1_ score of 0.79. We automatically extracted and visualized a range of features that characterise the manner in which the sows changed posture in order to provide comparative descriptors of sow activity and lying style that can be used to assess the influence of genetics or housing design. The methodology presented in this paper can be applied in large scale deployments with potential for enhancing animal welfare and productivity on farm.

## Highlights

• Data from a body-mounted accelerometer were used to classify posture and detect posture transitions in farrowing sows.
• Comparative descriptors of lying behaviour can be extracted allowing further in-depth analysis.
• The method may be used to predict the onset of farrowing and movements patterns potentially dangerous to piglets.

## 1 Introduction

Automatic classification and quantification of postures and posture transitions in domestic animals has substantial potential to enhance their welfare and productivity. Freedom of movement was one of the original “Five Freedoms” in the Brambell Report on farm animal welfare (Brambell, 1965), and the ability of housing systems to deliver this in a species-relevant way is a key component in modern welfare assessment schemes (Blokhuis et al., 2010). Changes in posture and activity may also be indicative of impending health problems (Szyszka and Kyriazakis, 2013, Weary et al., 2009). In the case of the domestic sow, automated posture assessments may facilitate the identification of additional specific behaviour traits that may confer advantages or disadvantages to the production system. Detection and analysis of activity patterns preceding farrowing may indicate the timing of parturition and the need for human intervention. Since the sow poses a significant crushing risk to the piglets (Marchant et al., 2001, Pitts et al., 2002, Špinka et al., 2000, Wechsler and Hegglin, 1997), the way in which she lies whilst in farrowing accommodation relates to her maternal ability and the adequacy of the housing provision, and has consequences towards the survival of her piglets.

Selection in swine production has resulted in a change of body shape leading to changes in the amount of control a sow can exhibit during lying (Marchant and Broom, 1996). Consequently, many piglets are at risk of being crushed either as the sow lies down (standing to lying event) or when she moves from lateral side to lateral side (rolling event). As an approach to accounting for this, the prevalent housing system for farrowing sows confines them to farrowing crates that restrict their movement, increasing survival rates of piglets by effectively minimising the risk from crushing (Cronin and Smith, 1992). Given that there is substantial individual variation in sow activity and lying behaviour (Marchant et al., 2001, Pitts et al., 2002, Špinka et al., 2000, Wechsler and Hegglin, 1997), categorisation of sows according to their lying behaviour and their genetic selection on the basis of this, could be used to improve the welfare and productivity of the farrowing system. With several sows farrowing at any one time in typical pig units, and potentially large numbers of animals required to perform genetic selection for aforementioned traits (Klein et al., 1973), automated assessment methods are a necessity for large scale utilisation of quantitative posture information.

The objective of this paper was to describe a methodology for automated posture assessment in sows around and during parturition. A tri-axial accelerometer attached to the monitored animal was used to collect relevant data. Accelerometers have previously been used to detect oestrus (Cornou, 2006), sow posture (Cornou and Lundbye-Christensen, 2008) and sow activity before, during and after farrowing (Cornou and Kristensen, 2014, Oczak et al., 2015), as well as the onset of parturition in sows (Cornou and Lundbye-Christensen, 2012). Prior work has revolved predominantly around posture state observations and activity levels (Cornou and Kristensen, 2014, Cornou and Lundbye-Christensen, 2012, Cornou and Lundbye-Christensen, 2010, Cornou and Lundbye-Christensen, 2008, Cornou et al., 2011, Oczak et al., 2015); by considering the periods in which a sow is moving between posture states, we can make valuable assessments of the lying style. Thus, the novelty of our work lies in:

1. The placement of the sensor, which allows the collection of data relating to potentially dangerous posture transitions as described in the literature.
2. Automation of the detection of posture transitions for the first time.
3. Automating the assessment of sow movements specifically targeting the prediction of behaviours dangerous for offspring.

In the first instance, we apply our methodology to a small number of six sows. Based on the outcome, the intention would be to use the method in large scale deployments for the automated screening of sows.

## 2 Materials and methods

### 2.1 Data collection

Six hybrid sows from the Newcastle University Cockle Park Pig Unit, due to give birth concurrently as part of a batch, were selected for assessment and moved to farrowing accommodation five days prior to their expected farrowing. The sows had all farrowed at least once previously, being either in their second or third parity. They were housed in standard farrowing crates with a concrete floor at the front and cast iron slats at the rear. All pens contained an area for the piglets in a front corner, with a heat lamp (alternately front left or front right corner, three of each). Motion data were recorded from each sow around the expected period of parturition in order to detect posture and posture transitions occurring both before parturition and also in the presence of piglets. Data collection was scheduled to run for four full days; however, the amount of data collected was reduced for two of the sows, due to problems with the sensor. A summary of the dataset can be seen in Table 1. The onset of parturition varied between sows.

#### 2.1.1 Sensing protocol

**Fig. 1:**
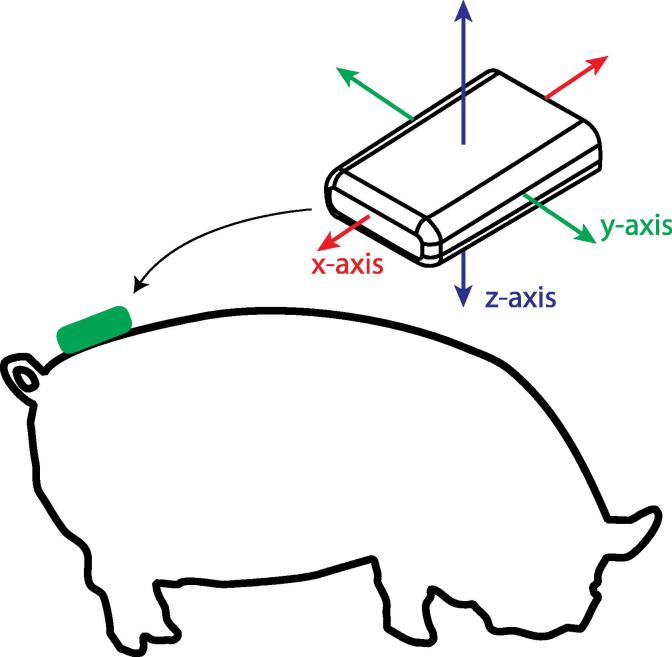
Positioning of sensor on the body of the sow. The orientation of the sensor is shown in relation to the sow. The sensor was secured to the hind-end of the sow between the hip bones and the tail-head. The x-axis of the sensor maps to the craniocaudal axis, the y-axis maps to the mediolateral axis, and the z-axis maps to the dorsoventral axis of the sow.

An Axivity AX3 logging accelerometer (Axivity, 2014) was attached to each sow using a combination of adhesive tape and glue according to a predefined protocol. All sows were shaved and cleaned in a small region between the tail-head and hip bones. The sensor was wrapped in duct tape and attached to the shaved area on the sow with strong double-sided carpet tape, ensuring a consistent sensor orientation. A coating of Evostick™ Instant Contact Adhesive was applied in a 2-in. area around the sensor with several strips of adhesive tape covering the glue and sensor for protection. The sensor collected motion data measuring between ±8g_0_ in the three spatial dimensions and sampled at 100 Hz. Attaching the sensor in the region between the tail-head and hip bones (Fig. 1) provided data relating to the forces associated with posture transitions that have been shown to pose the most danger to the piglets (Blackshaw and Hagelsø, 1990), particularly ‘flopping’ - a standing to lying transition that involves a rapid vertical displacement of the hind-end of the sow (Baxter et al., 2011).

#### 2.1.2 Annotation

In order to provide a verifiable record of the movements of the sows, independent from the sensor data, the sows were filmed continuously for approximately four consecutive days after placement in the crates. The camera was mounted above and to the rear of the pen and images were captured using Geovision software on a PC. Due to the restrictions on movement imposed by the farrowing crate, the full range of sow motion was visible in the recording. There was a small amount of variation in the length of the videos due to the timings at which the video footage was retrieved. In the case of Pig072, a section of footage was corrupted for the final seven hours of the study. Video footage was annotated using the open source application ELAN (Brugman et al., 2004). Accelerometer data were synchronized with the footage, allowing the annotations to be associated with the data directly (Plötz et al., 2012). The footage was annotated both for transitions between postures, and posture state. The annotations described the start and end of each posture transition, and the start and end of the periods in which the sows were in a consistent posture state.

### 2.2 Detection and segmentation of posture transitions

**Fig. 2:**
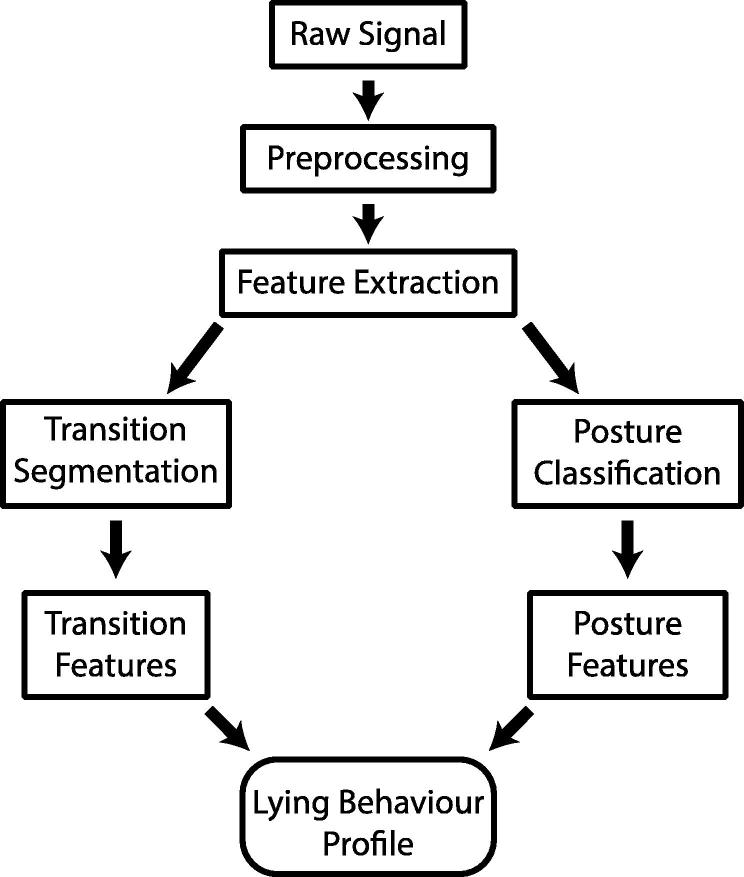
An overview of the processing workflow.

An analysis workflow was developed in order to automatically determine the periods within the data in which the sow transitioned from one posture to another (Fig. 2).

#### 2.2.1 Preprocessing and feature extraction

Static acceleration, i.e. the acceleration of the sensor due to the effect of earth’s gravity, was estimated from the raw signal using a moving average filter. The output from this process was used for detection of transitions and classification of posture, limiting the effect noise has on the analysis later in the workflow. Given raw signal samples $s(t)\in R^{3}$, with $-8⩽s_{i}(t)⩽8$, for each spatial axis *i*, the static acceleration was calculated according to Eq. (1):

(1) $$g(t)={\left(g_{x}\;g_{y}\;g_{z}\right)}^{T}\;\text{with}\;g_{i}(t)=\alpha \cdot s_{i}(t)-(1-\alpha )\cdot g_{i}(t-1)\text{;}\;i\in \{x\text{,}y\text{,}z\}$$

where α is a weighting component used to vary the amount that the signal compensates for rapid changes in acceleration (Swikatek et al., 2013).

For further analysis, the data were divided into short, continuous windows (frames) covering two seconds of sensor readings, i.e., 200 samples at 100 Hz. Three descriptors (features), summarizing the data in the frame, were then extracted for each frame to describe sow posture: pitch, roll and level of activity.

Pitch and roll, when calculated from static acceleration, describe the orientation of the sensor in 3-dimensional space. Pitch represents rotation of the sensor back and forth around the mediolateral axis, and changes when the pig lowers its hind-end to sit or lie. Roll measures change in rotation of the sensor to the left and right in relation to the pig, and varies as the pig moves between lying on either side. Given alignment as shown in Fig. 1, the z-axis follows the dorsoventral direction, the y-axis follows the mediolateral direction and the x-axis follows the craniocaudal direction. Using the estimation of the static acceleration g(t) (Eq. (1)) pitch *p*, and roll *r* were estimated as follows:

(2) $$p=\text{arctan}\left(\frac{g_{y}}{\sqrt{g_{x}^{2}+g_{z}^{2}}}\right)$$

where p∈R:]-90…90[. Pitch represents rotation around the mediolateral axis.

(3) $$r=\arctan\left(\frac{-g_{x}}{g_{z}}\right)$$

where r∈R:]-180…180[. Roll represents rotation around the craniocaudal axis.

The final feature calculated from the data was the standard deviation of the magnitude of the signal (within a frame) and was used as measure of the level of activity. The time-series describing the magnitude of the signal, $m=\{m_{1}\text{,}m_{2}\text{,}\ldots \text{,}m_{T}\}$, where *T* is the number of samples in the frame, was calculated as below for each sample in the sub-frame:

(4) $$m(t)=\sqrt{x{\left(t\right)}^{2}+\text{y}{\left(\text{t}\right)}^{2}+z{\left(t\right)}^{2}}$$

The level of activity for the whole frame was then calculated as:

(5) a=σ(m)

where *σ* denotes the standard deviation.

#### 2.2.2 Transition detection and segmentation

Feature extraction resulted in three features for each frame of data f(i)={p,r,a} Posture transitions were detected by observing changes in the pitch and roll of a particular sensor that exceed empirically determined thresholds *θ_p_* for pitch, and *θ_r_* for roll. To provide a more robust assessment of the change in posture, each frame f(i) was combined with the five subsequent frames into larger 12 s frames with an overlap of 60%.

For this new set of extended frames across the session, the frames f(i) and f(i-1) were considered to describe a transition point if the below rule holds:

(6) $$|f{\left(i\right)}_{p}-f{\left(i-1\right)}_{p}|>\theta_{p}\vee |f{\left(i\right)}_{r}-f{\left(i-1\right)}_{r}|>\theta_{r}$$

Frames adjacent to the transition point were also required to satisfy the above rule to ensure that a transition had occurred, rather than a temporary adjustment of position. The actual point of transition was considered as the mid-point between the frames in which the threshold was initially exceeded.

**Fig. 3:**
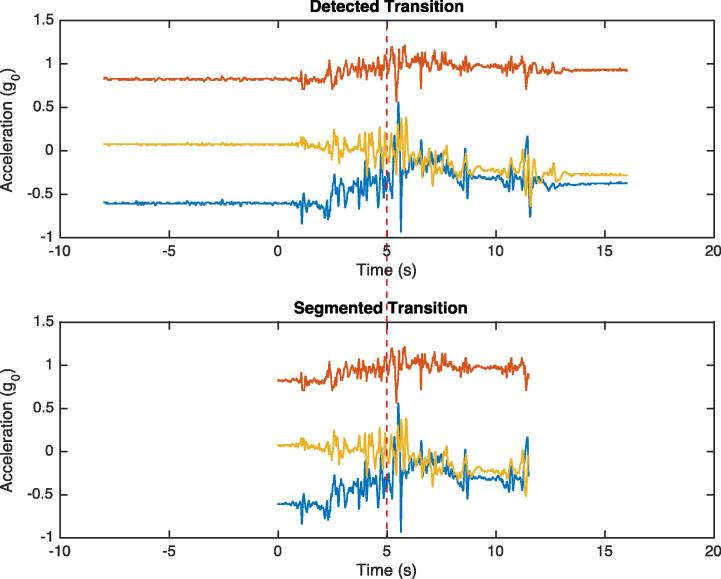
An example segmentation of a posture transition. The transition point occurs at five seconds, indicated by the dashed red line. By performing a search for stabilization of the features either side of this point, the start and end of the transition is identified. (For interpretation of the references to colour in this figure legend, the reader is referred to the web version of this article.)

Segmentation of the transitions was performed by identifying the point in the data either side of the transition at which the pitch and roll stabilized. In order to accomplish this, the change in pitch and roll was evaluated starting from the transition point. The start and end point of the transition was marked where the sensor was no longer in a consistent orientation for 1.5 s. The result of this process is illustrated in Fig. 3.

### 2.3 Posture classification

In order to classify sow posture automatically, a Support Vector Machine (SVM) classifier using a Radial Basis Function (RBF) kernel, was trained on the frames of feature data (Schölkopf and Smola, 2002). Parameters were optimized using standard sequential minimal optimization (SMO) and hyper-parameters were optimized using grid search. The classifier processed feature vectors extracted from the shorter, 2-s frames, and predicted class (posture) associations for each frame. A sow can be in one of five, mutually exclusive postures at any time: Standing (ST), Sitting (S), Lying on left hand side (LL), Lying on right hand side (RL), and Sternal Lying (SL). Kneeling, a posture in which the sow rests on her front knees with her hind end raised, was considered to be a transitory posture and thus not given a distinct class.

### 2.4 Transition feature extraction

A set of descriptive features were identified which characterise each transition:

i. The duration of the transition described the amount of time the sow spent between posture states, and thus relates to the speed with which the transition was made.
ii. The peak acceleration, as described by the maximum value of the magnitude of the signal recorded during a transition, considering all axes together.
iii. The range of acceleration described the point in the transitions when the largest change from acceleration to deceleration took place and calculated for each of the three acceleration axes. The maximum of these three is used, representing the largest change in acceleration in any one direction.
iv. The rate of change of the acceleration of the sensor, known as the ‘jerk’. When a sow lowers herself to the ground, her hind-end may come to a stop suddenly or gently. The former will produce a large deceleration value, and similarly the jerk will be large. In the latter case, the deceleration is the same, however occurs over a longer period of time, producing a lower value for jerk. Jerk is defined formally as the first derivative of acceleration.
v. The rate of change of both pitch and roll were used to define the smoothness of the transitions. In contrast to jerk however, this pair of features is calculated based on preprocessed data, rather than on the raw values. These features are calculated as the first derivative of pitch and roll.

### 2.5 Lying behaviour profiles

In addition to extracting features based on the individual transitions, characteristics of the datasets relating to the lying behaviour of each sow throughout the period in which the sow was under observation were extracted. Key amongst the descriptors extracted was the quantification of how each sow preferred to lie. By collating the results of the posture classification for each sow, a time budget was produced which outlines the proportion of time the sow spends in each posture. The frequency with which the sow changes its orientation was used as a robust indicator of the level of activity at a higher level than the features used for signal analysis. This was performed by taking a moving average of the number of transitions in each two-hour period, in increments of 12 min.

### 2.6 Evaluation and validation

#### 2.6.1 Transition detection and segmentation

The output of the detection and segmentation process is a 2-dimensional vector of timestamps for each transition indicating the start and end of the transition. An event based evaluation was performed to determine true positives (*TP*) false positives (*FP*), and false negatives (*FN*). The algorithm is considered to have achieved a true positive detection when the detected transition coincides with a labelled transition, according to the annotated ground truth. If more than one detection coincides with the annotation, it is considered as a single correct result; however, this is rare within the data. A false positive occurs where a detected transition does not lie within any annotation. Based upon these results, three standard measures for prediction evaluation were calculated: precision, recall and *F*_1_ score. Precision describes the fraction of the transitions predicted as positive that are actually positive:

(7) $$\mathrm{Precision}=\frac{\mathrm{TP}}{\mathrm{TP}+\mathrm{FP}}$$

Recall describes the fraction of all transitions that are predicted as positive:

(8) $$\mathrm{Recall}=\frac{\mathrm{TP}}{\mathrm{TP}+\mathrm{FN}}$$

The *F*_1_ score is the harmonic mean of precision and recall and reflects both in an intuitive manner:

(9) $$F_{1}=2\left(\frac{\mathrm{recall}\times \mathrm{precision}}{\mathrm{recall}+\mathrm{precision}}\right)$$

#### 2.6.2 Posture classification

In order to evaluate the classifier a “leave-one-pig-out” approach was employed, in which the classifier was trained sequentially using five of the six sows, and tested on the sixth. The mean of the evaluation measures across the six experiments was taken. This gave a measure of the classifiers ability to accurately predict posture on unseen data.

#### 2.6.3 Transition features and lying profile

In order to evaluate the suitability for comparison of the features selected for analysis, the Empirical Cumulative Distribution Function (ECDF) of each feature was calculated. The value produced by the ECDF function (*x*) is equal to the proportion of transitions that produce a feature value lower than, or equal to *x*. The shape of the distribution function was used to compare the variation of the feature distribution between sows. By calculating the ECDF of a feature using transitions from all sows, a baseline is established. The EDCF of the features for each sow individually was calculated and compared with the baseline.

## 3 Results

### 3.1 Transition detection and segmentation

The dataset recorded contained a total of 1268 transitions. Of these, 965 were correctly identified (true positives), whereas 303 were not detected (false negatives). In 204 of the 12-s frames the data were incorrectly labelled as being a transition (false positives). The precision and recall values were 0.826 and 0.761 respectively. Considering these two results, the *F*_1_ score was 0.792. The precision value of 0.826 demonstrates that less than one fifth of the positive predictions were incorrect. A recall value of 0.761 shows that less than a quarter of actual transitions were missed. We did not weight either precision or recall above the other, consequently the *F*_1_ score gives a representative combination of the two metrics.

### 3.2 Posture classification

**Fig. 4:**
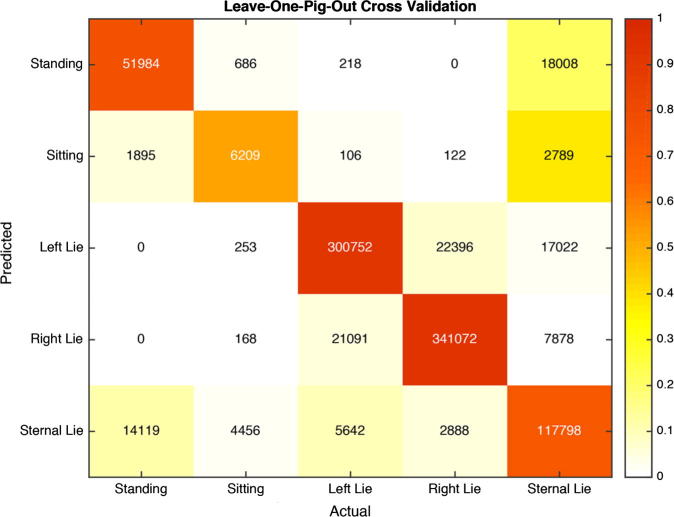
Confusion matrix outlining the results of predicting posture using a Support Vector Machine (SVM) classifier. The colour represents the proportions of the class that were predicted in relation to the number of instances of that class, a darker red is considered a better result. The number inside the cell denotes the number of instances that were classified according to the labels on the axes. A strong dark series of cells diagonally across the matrix reflects accurate classification, as these cells show the proportion of correctly predicted frames. Darker cells outside of the main diagonal describe a higher proportion of frames misclassified. (For interpretation of the references to colour in this figure legend, the reader is referred to the web version of this article.)

The mean *F*_1_ score for the classifier across all classes was 0.776. A class-by-class breakdown of results is shown in Table 2. Fig. 4 provides a visual description of the classifiers results. Of the five classes, *F*_1_ scores for all but one class are over 0.74, however, the *F*_1_ score for the sitting class is lower at 0.542. This indicates a higher degree of confusion between sitting and the other classes. The confusion matrix (Fig. 4) shows that the misclassifications of the sitting class were mostly classified as Standing and Sternal Lie.

### 3.3 Transition feature extraction

**Fig. 5:**
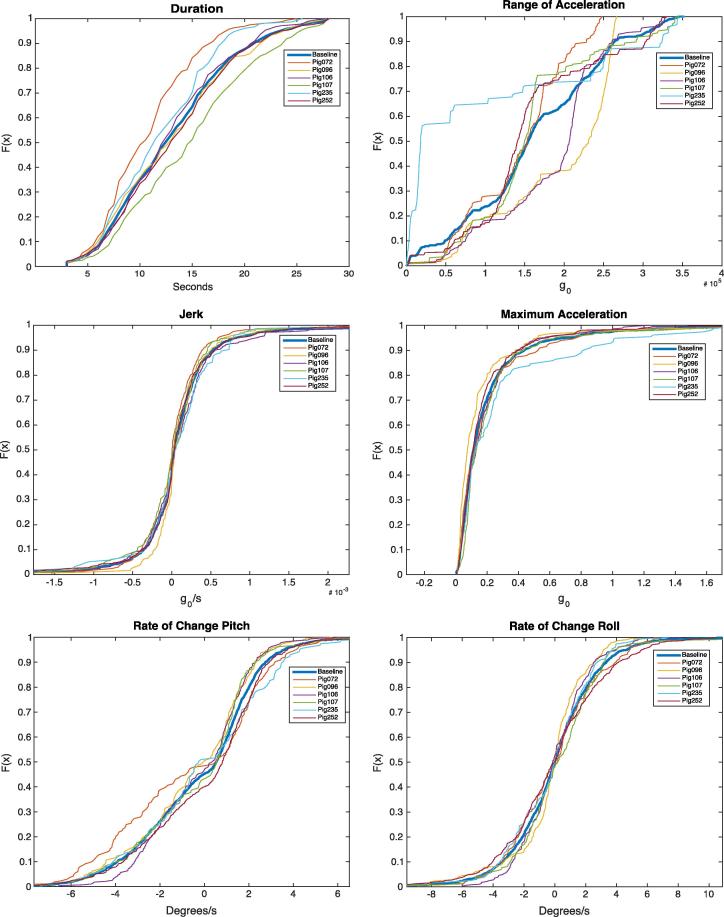
Empirical Cumulative Distribution Function (ECDF) plots for descriptive features extracted from posture transitions. ECDF plots show the cumulative proportion of instances on the vertical axis plotted against the feature value on the horizontal axis. The thick blue line describes the ECDF of all transitions across all sows. The other lines represent the ECDF plots for each individual sow. Note the cyan line in the range of acceleration from Pig235, which describes a distribution containing a transitions with smaller ranges of acceleration values, whilst the distribution for the maximum acceleration shows that larger accelerations are more common than in the other sows. Best viewed in colour. (For interpretation of the references to colour in this figure legend, the reader is referred to the web version of this article.)

Fig. 5 shows the ECDF plots for the six features described in Section 2 i.e., transition duration, range of acceleration, jerk, maximum acceleration, rate of change of pitch, and rate of change of roll. It can be seen, for example, that a particularly inactive sow (Pig235) produced noticeably different feature distributions for several of the features. The ECDFs were generated from the underlying distribution of the feature values that were the primary output of the algorithms. The plots can be interpreted to assess the behaviour of the sows across all transition events, and also provide information regarding the shape of the feature distributions. A good example of this is in the ECDF plot for the duration feature. It can be seen that the curve for Pig072 lies well above the baseline – which described the distribution across all transitions for all sows - suggesting that this sow displays a larger proportion of short duration transitions. Conversely, the curve for Pig107 lies underneath the baseline curve, indicating this sow displays a larger proportion of long duration transitions. Of particular interest is Pig235, which farrowed within the first 6 hours of the study. This sow had a markedly different shaped distribution for both range of acceleration and for maximum acceleration. The curve for the maximum acceleration feature lies below the baseline, indicating that the sow displays a larger proportion of transitions with a higher maximum acceleration. The curve for range of acceleration lies noticeably above the baseline curve, indicating a larger proportion of the transitions have a small range of accelerations.

### 3.4 Lying behaviour profiles

**Fig. 6:**
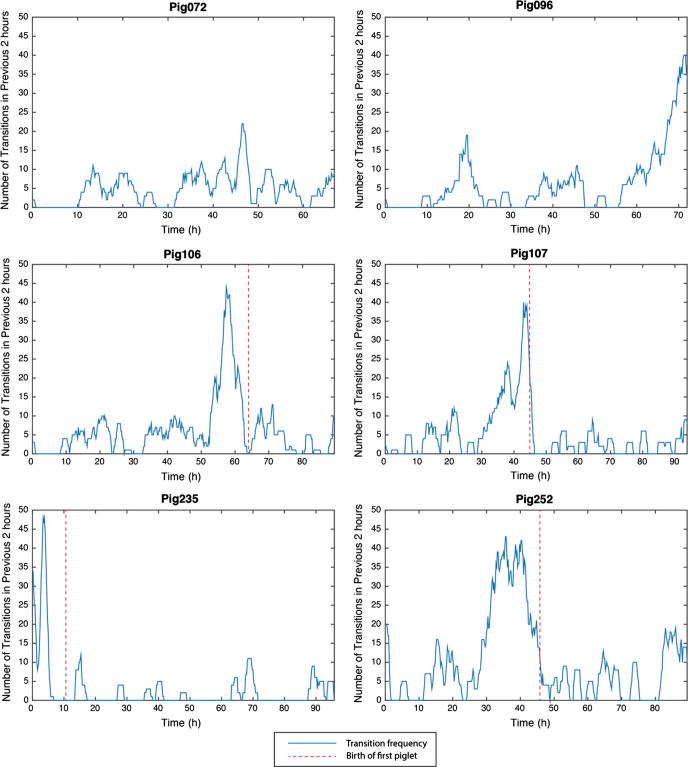
Posture change frequency. A moving average is taken over a 2-h period in increments of 12 min. The large spikes shown in the charts for Pig106, Pig107, Pig235 and Pig252 coincide with the increased activity in the build up to farrowing. The dashed red line indicates the period in which the first piglet was born. Pig072 and Pig096 did not farrow whilst the sensor was recording, although Pig096 farrowed shortly after the sensor stopped. (For interpretation of the references to colour in this figure legend, the reader is referred to the web version of this article.)

A visualisation of the transition frequency data can be seen in Fig. 6. These charts provide a representation of the sows’ activities throughout the recording periods.

## 4 Discussion

Piglet neonatal mortality due to crushing by the sow has been a long standing problem in modern farrowing systems (Baxter et al., 2011, Edwards, 2002); it represents a welfare and productivity issue. Historically, it has been reduced by confining the sow to a crate, thus minimising her movements and providing safe areas for the piglets (Cronin and Smith, 1992). However, even in these environments, sows exhibit nesting behaviour. Current research suggests that if nesting behaviour has not been carried out to the satisfaction of the sow, nest building may continue into parturition, increasing the amount of risk presented to the piglets (Damm et al., 2000). Identifying housing conditions that allow appropriate expression of this behaviour is therefore a research need. Furthermore, if posture changing behaviour can be characterised, it may be possible to select sows that demonstrate ‘advantageous’ lying behaviour that minimises the risk to her piglets and/or to intervene at time points when problems are most likely to happen. Currently, lying quality and activity of sows is measured visually and temporally, through live observation or videos and recording the latency to complete each of the stages of lying (Marchant et al., 2000). In order to fully understand lying behaviour we need to be able to measure not only the time taken for a sow to lie, but also the accelerations involved in the process.

The objective of this paper was to develop a methodology for automatic quantification of posture and posture transitions of sows around, and during the period of parturition. This has been achieved through analysis of data collected from a study with six sows, with over 525 h of accelerometer data. Posture changes were automatically detected with an accuracy comparable to similar studies, considering the different classes under observation. Features quantifying motion characteristics of the transitions have been identified and a framework for their extraction and comparison was developed. Although the experiment was conducted on only six sows, the outcomes demonstrate the potential of the approach to be applied to large scale deployments.

The use of accelerometer data to record the movement of animals is well established, however in the design of this study other options were considered. “Computer vision” describes a set of techniques that enable automatic interpretation of visual data to be conducted (Forsyth and Ponce, 2003). There are, however, specific issues that preclude the application of computer vision techniques in the context of this study. Primary amongst these was the study’s aim of generating detailed representations of posture transitions and the associated forces. Without biometric measurements specific to the individual sows, it is not possible to measure force. However, insights can be gained into the forces exerted during transitions from the acceleration data as force and acceleration increase proportionally. It could be argued that the inclusion of a gyroscope in the sensing platform would have been appropriate. Due to the power requirements of a gyroscope, however, (depending on the device, up to 20× the requirements of an accelerometer (NXP Semiconductors, 2006, STMicroelectronics, 2010)), the sensor would have been unable to record for the intended duration of the study, making this unfeasible.

Given the requirement that detailed information relating to the accelerations exhibited during posture transitions should be generated, the positioning of the sensor was a key consideration. Previous studies investigating the classification of posture and activity in farrowing sows have employed sensors attached to the animals’ crates, collars and legs (Cornou and Lundbye-Christensen, 2008, Huang et al., 2004, Ringgenberg et al., 2010). Collar mounted sensors have limited capacity to accurately observe posture changes at the hind-end of the sows, consequently the decision was made to position the sensor on the sow’s hind-end, above the tail-head and below the hip bones. Such an approach to quantifying the characteristics of posture transitions has not been reported previously in the literature, and as such provides an entirely novel perspective for this kind of analysis.

The precision and recall values for the segmentation algorithm indicate that a sizable proportion of posture transitions were correctly identified. However, these results also demonstrate that there is scope for improvement in the system. A portion of the false positive results were produced when the sensor recorded a large movement of the sow away from its current position, in order to scratch or shift temporarily for example, before returning to its original posture without changing posture classes. Where a sow’s posture following the movement was sufficiently different to the original posture (although not in a concretely different posture category) the segmentation algorithm was prone to recording a false positive detection. Misdetections were also produced by very gradual changes of posture over a period of several minutes. These transitions occur mainly between sternal and lateral lies, although the data set includes transitions between a sit and a lie where this also occurs. The failure to detect the transition occurs due to the orientation of the sensor consistently changing by amounts lower than the thresholds used in Eq. (6), resulting in false negative results. Future work would involve eliminating these causes for failure. This could be achieved through the use of an additional sensor in a different location on the pig to verify posture changes; however, this would necessarily increase the complexity of the system and would require a different approach.

There is a body of work that focuses on behaviour analysis of farrowing sows, and in particular the use of motion sensing platforms to perform activity recognition, although very little attention is given to assessment of posture transitions, outside of our work. For example, Cornou and Lundbye-Christensen (2010), considered activity recognition through the use of accelerometer data in farrowing sows as a two class problem, lying vs active, and report an accuracy of up to 97%. Marchioro et al. (2011) employed an heuristic based approach to classify activity states in sows, where the states were classified as: lateral lying, sternal lying, medium activity and high activity. Through the use of a rule based approach using a measure for activity taken from each axis on a per sample basis, precisions of “up to 90%” were reported, although figures as low as 81% are also given for different classes, and no mention is made of the recall values for these classifications. This highlights that their algorithm provides a low number of false positive results but does not take into consideration positive results classified negatively. Cornou et al. (2011) employed a Multi-Process Kalman Filter developed in prior work (Cornou and Lundbye-Christensen, 2010) to classify sow activity. Classification accuracies of between 75 and 100% were achieved. These results could be considered to be stronger than those presented in this study, however, it can be argued that the broader classes chosen for assessment in those studies are a key contributing factor.

Escalante et al. (2013) presented an overview of a range of machine learning approaches to classifying sow behaviour, using the same data set described in Cornou and Lundbye-Christensen, 2008, Cornou et al., 2011. They described a five class problem and aimed to differentiate between Feeding, Rooting, Walking, Sternal Lying and Lateral Lying. The top performing classifier tested, logitboost, correctly classified an average of 74.64% 1-s observations. When considering 2-min series, the logitboost classifier averaged 80% of series accurately classified. Whilst Escalante et al. (2013) do not provide the *F*_1_ scores for the logitboost classification, they can be calculated by considering the number of series tested as well as the percentage correctly predicted. Calculating the *F*_1_ score in this way provides an un-weighted mean *F*_1_ of 0.59, significantly lower than that produced by the SVM classifier employed in this paper.

Based on the range of movement available to sows housed in farrowing crates, the classes chosen for this work provide a detailed characterisation of the posture of the sow. Whilst altering these classes to simplify the problem could produce better classification performance, it was felt that to be complete the system should classify to as detailed a level of posture as possible. Given that this is the case, however, specific drawbacks were encountered. Fig. 4 shows that the posture classification system developed in this work robustly identified periods in which the sow was lying laterally. It can be seen, however, that classification between Sternal Lying and Standing as well as between Sternal Lying and Sitting was problematic. Sitting can be seen to be a largely transitory state i.e., the sow sits for a short period between postures. This is similar in nature to the Kneeling posture, however, due to the increased frequency and duration compared to Kneeling, it was decided to assign Sitting a discrete classification. Despite this, the proportion of the data in which the sows are in the Sitting posture was substantially lower than the other postures, and as such there was considerably less data with which to train the classifier for Sitting.

Ringgenberg et al. (2010) attempted to classify sitting behaviour and also found this to be a particularly difficult class to predict, correctly predicting only 37% of sitting postures. In their work, sensors were secured to a hind leg in addition to the sows’ backs for a period of 6 h. This approach produced very good results for classifying Standing (99.6%) and Sternal (93.5%) and Lateral Lying (96.7%), however, for longer term deployments this might be unsuitable as the sensors would likely be removed by the sows, as they described. Cornou et al. (2011) conflate Sitting, Standing and Sternal Lying into a “Medium activity” class, which again produces the least compelling results of the classes identified. Mainau et al. (2009) described a photoelectric implementation of a Standing to Lying Sensor. They reported an inability to distinguish between sitting and standing and, as such, again merged it into a single class with the Standing posture. This highlights the difficulties associated with correctly predicating this class, and identifies an open problem for further investigation and improvement. An approach to improving this would almost certainly rely upon the addition of further sensors. The involvement of sit-lie/sit-stand transitions in piglet crushing is generally considered to be less than the involvement of stand-lie transitions (Marchant et al., 2001, Weary et al., 1996, Weary et al., 1998). Nevertheless, Vieuille et al. (2003) reported that 27% of crushing events occurred during a sitting transition and, as such, it could be argued that accurate classification of sitting would be essential for a complete system.

The transition frequency plots show the cumulative amount of transitions for each 2-h period, providing clear descriptions of the level of activity exhibited by the sow. As can be seen in Fig. 6, the transition frequency plots from Pig106, Pig107, Pig235, and Pig252 clearly show the dramatic increase in activity as the sows attempt to exhibit nest building behaviour. Manual checks of the video recordings showed that these spikes in activity occur in the 12–18-h period before the onset of farrowing. The peak rate of positional changes has been reported to occur in the 6 h prior to the onset of farrowing (Mainau et al., 2009), although 12 h prior to farrowing has also been suggested (Huang et al., 2004). In other work in the field, activity data similar to this have been used to predict the onset of farrowing (Marchioro et al., 2011, Oczak et al., 2015) and, given a larger dataset, it would certainly be possible to use the transition frequency metric generated in this study to predict farrowing. An accurate prediction of the onset of farrowing would provide farmers the ability to provide timely neonatal piglet care without reliance on continual monitoring. Detection of prolonged farrowing, in particular, can be used as an indication that human intervention is required (White et al., 1996, Zaleski and Hacker, 1993).

Baxter et al. (2011) showed that a key factor towards distinguishing sow selection lines more prone to crushing appears to be care in movement. This suggests that there might be a genetic component in the sow’s propensity towards crushing her piglets and that it may be possible to select against such behaviours (Wechsler and Hegglin, 1997). The method we present in this paper would be useful in determining the degree to which the posture change traits associated with crushing in sows are exhibited, and would consequently allow for selection of production sows based on these traits. Genetic selection of livestock for specific traits requires large numbers of individuals characterised both for the traits and their pedigree (Klein et al., 1973). Such large numbers can only be achieved through an automated monitoring of the relevant traits.

Further development of the systems described in this work should consider the choice of features extracted from the transitions. Refinement, or expansion, of the features selected would allow for different analyses to be conducted. The features used in this study focus on describing the control, or lack thereof, exhibited in the sow’s lying behaviour. Other analyses could be employed to assess the frequencies involved in transitions, the behaviour before and after the transitions, or the spatial context of the transitions. The algorithms developed for this study have been designed to be easily extensible should these analyses be required.

## 5 Conclusion

We have presented a framework for the automated detection and assessment of sow posture change, as well as an approach for sow posture classification through the analysis of inertial measurements. Through this, we have provided access to data relating to the characteristics of sows’ lying behaviour during parturition. Such data could prove to be invaluable for several different applications, notably in characterising the nesting activity of sows’ before and during farrowing, and in identifying sows with a predisposition towards posture changes that pose a danger to new-born piglets post-partum. Although there were some issues with the detection of specific postures such as sitting, large scale application of the method and modifications identified above should improve its utility. The algorithms described herein have been designed with extensibility in mind from the outset. The implementation of further behavioural features can be conducted and integrated simply and quickly, allowing for rapid assessment of lying behaviour with objectives different from those identified in this study.

## Ethics

The experiments were performed at Newcastle University, Cockle Park farm. Attaching the accelerometer on the sows was the only deviation from normal husbandry procedures. The study was approved by the Newcastle University Animal Welfare Ethics Review Board.

## Figures and Tables

**Table 1:** Dataset description for the study. Six sows were used and in each case the intention was to record sensor data for four days. In the case of Pig072 and Pig096 the sensor did not record for the full duration, see notes for explanation.

Sow ID	Video duration [h]	Sensor data duration [h]	Notes
Pig072	89	69	*Sensor fell off*
Pig096	95	74	*Sensor failed*
Pig106	95	95	
Pig107	96	96	
Pig235	96	96	
Pig252	96	96	

**Table 2:** *F*_1_ results produced by the Support Vector Machine (SVM) classifier when validated using a leave-one-pig-out (LOPO) cross validation technique. LOPO cross validation refers to the process of training the classifier on data from five of the six sows and testing it on the sixth. This process was repeated, leaving each sow out in turn. The classification results for each test set were then combined to produce results for the full data set.

Posture	Number of frames	*F*_1_ score (LOPO)
Standing	70 896	0.749
Sitting	11 121	0.542
Left lie	340 423	0.900
Right lie	370 209	0.926
Sternal Lie	144 903	0.764
Mean		0.776
